# Analysis of Glioblastoma Multiforme Tumor Metabolites Using Multivoxel Magnetic Resonance Spectroscopy

**Published:** 2020

**Authors:** Meysam Siyah Mansoory, Ayob Faramarzi, Karim Khoshgard, Hadi Mozafari

**Affiliations:** 1. Department of Biomedical Engineering, Faculty of Medicine, Kermanshah University of Medical Sciences, Kermanshah, Iran; 2. Pharmaceutical Sciences Research Center, Kermanshah University of Medical Sciences, Kermanshah, Iran; 3. Department of Medical Physics, Faculty of Medicine, Kermanshah University of Medical Sciences, Kermanshah, Iran; 4. Medical Biology Research Center, Kermanshah University of Medical Sciences, Kermanshah, Iran

**Keywords:** Glioblastoma multiform, Magnetic resonance spectroscopy, Neurochemical profiles, Voxel

## Abstract

**Background:**

Glioblastoma Multiforme (GBM) is the most common and deadly type of primary brain tumor in adults. Magnetic Resonance Spectroscopy (MRS) is a noninvasive imaging technique used to study metabolic changes in the brain tumors. Some metabolites such as Phosphocholine, Creatine, NAA/Cr, and Pcho/Cr have been proven to show a diagnostic role in GBM. The present study was conducted to analyze important metabolites using MRS multivoxel in GBM tumor.

**Methods:**

In this study, information was collected from 8 individuals diagnosed with GBM using Siemens multivoxel MRS with a magnetic field strength of 3 T. Data were obtained by Point-Resolved Spectroscopy (PRESS) protocol with TE=135 *ms* and TR=1570 *ms*. NAA, Pcho, Cr, Ala, Gln, Gly, Glu, Lac, NAAG, and Tau metabolites were extracted and evaluated statistically.

**Results:**

Given total number of normal voxels and total number of all voxels, levels of Cr, Glu, NAA, NAAG, and Gly/Tau ratio in healthy voxels were significantly higher than tumoral voxels (p=0.005, p=0.03, p<0.001, p<0.001 and p=0.041, respectively). In contrast, levels of Gly, Gln, Tau, Lac/Cr, Pcho/Cr, Pcho/NAA, Lac/NAA, and Gln/Glu ratios in tumoral voxels were significantly more than healthy voxels (p=0.001, p= 0.037, p<0.001, p=0.010, p<0.001, p<0.001, and p=0.024, respectively). However, levels of Lac and Pcho had no significant difference in the two types of voxels.

**Conclusion:**

In summary, compared to patients with glioblastoma with ^1^H-MRS, the Pcho/Cr and Pcho/NAA ratios, and NAAG are the most important parameters to differentiate between tumoral and normal voxels.

## Introduction

Glioblastoma Multiforme (GBM) is the most malignant and common tumor of central nervous system accounting for 40% of all types of brain tumors, among which 15 to 20% are of high grade 1,2. In 2017, more than half of 2607 patients with malignant brain tumor in the United States were diagnosed with GBM. Average survival time of patients with GBM under treatment and untreated patients is equal to 14.6 and 6.9 months, respectively, and the 5-year survival rate with treatment is reported as 9.8% 3.

MRI as a non-invasive method is a suitable technique used to diagnosis of GBM tumor providing anatomical visualization of the brain tumor. In MRI images, GBM is seen as ring-enhancing lesions. However, this pattern is not exclusive to GBM and is present in other diseases such as Tumefactive multiple sclerosis, abscess, and metastasis 4. In spite of high contrast of soft tissue in MRI images for GBM tumor site, spatial spread of cerebral malignant cells is not detectable with MR modalities 5. Distinction between healthy and tumoral areas is critical for treatment process including surgery, chemotherapy, or radiotherapy 6.

Detailed information of brain tissue is needed to determine precise spatial location of the tumor. Neurological disorders alter concentration of metabolites such as Phosphocholine (Pcho), Creatine (Cr), and N-acetylaspartate (NAA) in brain tissue as well as concentration ratios of like NAA/Cr and Pcho/Cr. Biochemical profile of brain tissue including concentration and proportion of metabolites can be measured by non-invasive Proton magnetic resonance spectroscopy (1HMRS) 7.

Using a magnetic resonance imaging system and some hardware arrangements along with a software platform, Magnetic Resonance Spectroscopy (MRS) data can be obtained to calculate brain metabolites. MRS is applicable not only for hydrogen (^1^H) but also on many other nuclei or isotopes like carbon (^13^C), nitrogen (^15^N]), fluorine (^19^F), sodium (^23^Na), and phosphorus (^31^P). Abundant elements like hydrogen are used in brain imaging 8.

The first MRS report on the human brain dates back to 20 years ago 9. MRS has been applied to evaluate various types of brain tumors and has been proven as a part of clinical evaluation of the tumor 10. Einstein *et al* showed that patients with stereotactic radiosurgery have a longer survival for (Pcho/NAA) >2.1 than standard treatments 11. Deviers *et al* found that concentration ratio of (Lac/NAA)>=0.4 in voxels prior to radiotherapy could be meaningfully used to predict recurrence of the tumor 12.

Haaga *et al* introduced important brain metabolites such as NAA, Pcho, Lactate (Lac), Cr, Glutamic Acid (Glu), and Glutamine (Gln). Among them, the NAA metabolite has a vital importance in neurological diseases, especially brain tumors. They also reported that NAA/Cr and Pcho/Cr ratios in GBM tumor decreased and increased, compared to healthy tissue of the brain, respectively 9.

Parra *et al* found that distribution of metabolites such as NAA and Pcho in healthy and tumoral voxels showed a significant difference. On the other hand, concentration of NAA decreased and concentration of Pcho increased in tumoral voxels compared to healthy ones 5. Analyzing MRS data, Crain *et al* found that five ratios of Pcho/Cr, Cr/Pcho, Lac/Pcho, Lac/Lip and Lip/Lac are useful to differentiate between GBM healthy and tumoral voxels 7.

Various techniques have been implemented to quantify concentration of metabolites in MRS signals, but quantifications are still accompanied with high variations and big errors 13. Recent studies showed that, seemingly there are unknown metabolites or ratios, which need to be evaluated for GBM tumor using MRS multivoxel data. In this study, such ratios and metabolites were investigated.

## Materials and Methods

**Figure 1.**
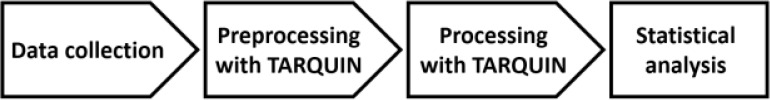
The study algorithm

General layout of this research is depicted in figure 1.

### Data collection

Data related to 8 patients with GBM were obtained at Imaging Center of Imam Khomeini Hospital in Tehran, Iran during March 2017 to March 2018. Before scanning, a complete explanation was given to all the subjects and ethical letters of consent were also signed. In addition, the data were obtained based on codes of the Medical Ethics Committee of Kermanshah University of Medical Sciences (Code of Ethics: IR.KUMS. REC.1397.142). Demographic information of studied patients is presented in table below.

In this study, imaging was performed through multivoxel Spectroscopy using a 3-Tesla MRI scanner made by Siemens Corporation. Multivoxel Spectroscopy was used to obtain a high-quality spectrum from a large area 14. The Point-Resolved Spectroscopy [PRESS] imaging protocol was used with following parameters: TE=135 *ms*, TR=1570 *ms*, and 1024 data points.

Magnetic field shimming was automatically performed before obtaining data. Long TE used for data collection in this study has several advantages over short TE: 1) in long TE, peak of lactate and alanine is better detectable than that of the fat compared to short TE and 2) NAA peak is distinguished from Glx (Glu or Gln) and the base line distortion reduces as well 15. Tagging and regionalization of tumoral and non-tumoral regions were performed using MRI data of the subjects by a radiologist at Imam Reza Hospital in Kermanshah, Iran.

### Pre-processing

**Figure 2.**
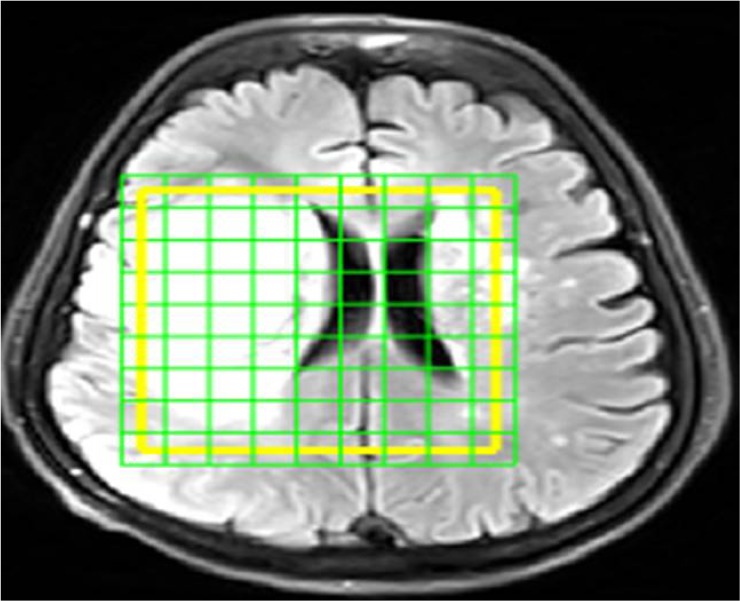
A sample of MRI image in SIVIC software in which the border of the tumor region at the time of imaging is determined as yellow box. Each green tagged square represents a voxel. The amount of each metabolite corresponding to each voxel is extracted in subsequent steps.

For pre-processing of the data, MRS range of imaging was determined using SIVIC; an open-source, standards-based software framework and application suite for processing and visualizing Digital Imaging and Communications in Medicine (DICOM), MR Spectroscopy data processing, and visualization. The MR image was subsequently overlaid on the MRS. Finally, the target region was determined (Figure 2).

The MRS signal obtained from device is a sinusoidal transient signal consisting of unwanted signals such as water signal, baseline signal, and residual signal (noise). The main signal received was defined as:

S[t]=Met[t]+w[t]+Res[t]

Where, S represents received signal, Met represents metabolites signal, w is water signal, and Res is residual signals including noise and other things.

**Figure 3.**
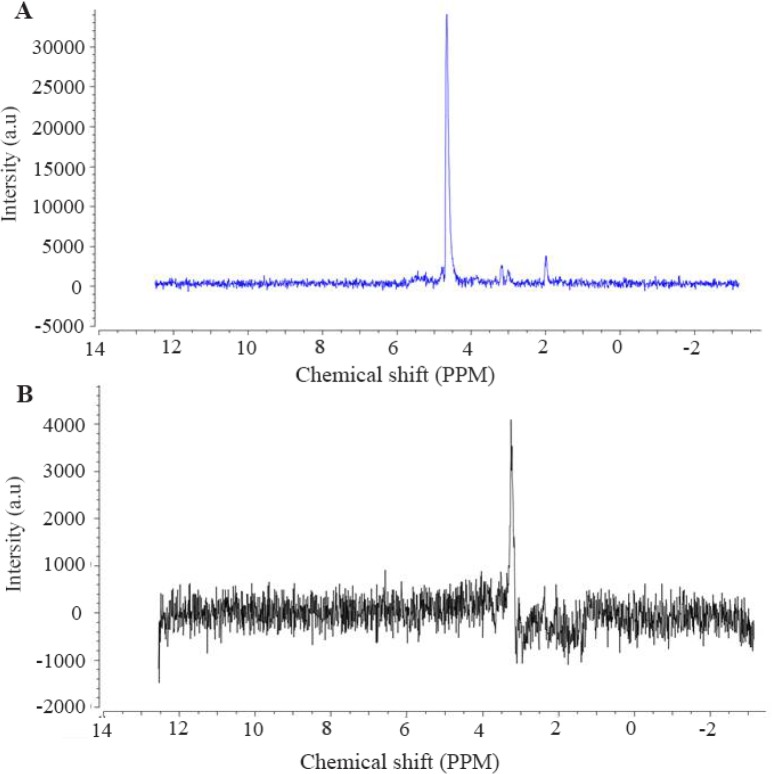
A) MRS signal information in a voxel before water suppression. As indicated, the high peak is related to water signal. The size of other metabolites is affected by the presence of water peak. The most important step in preprocessing phase is the removal of water signal. B) the signal obtained after the removal of the water peak. As each metabolite has a definite profile, the concentration of the metabolites is obtained from the peaks of this signal.

The baseline signal originates from macromolecules, appearing at early stages in signal samples, and then rapidly disappears. The background signal is smooth. Like other MR imaging techniques, various artifacts such as Poor Shimming, Phasing Errors, and Chemical shift displacement influence Res signal quality 16,17. The main goal of pre-processing stage is finding signal of metabolites among the main signal and quantify metabolites with acceptable accuracy. For this reason, since levels of metabolites in brain tissue is much lower than water, water signal, which is 10,000 times larger than other metabolites 18 must be suppressed. In the present study, water suppression was done with a frequency of 45 *Hz*. Figure 3A illustrates this step of pre-processing process.

Since the baseline signal has an undesirable effect on amount of other metabolites, to accurately calculate these values, the baseline signal should be reduced from the signal after water suppression (Figure 3B).

### Processing

**Figure 4.**
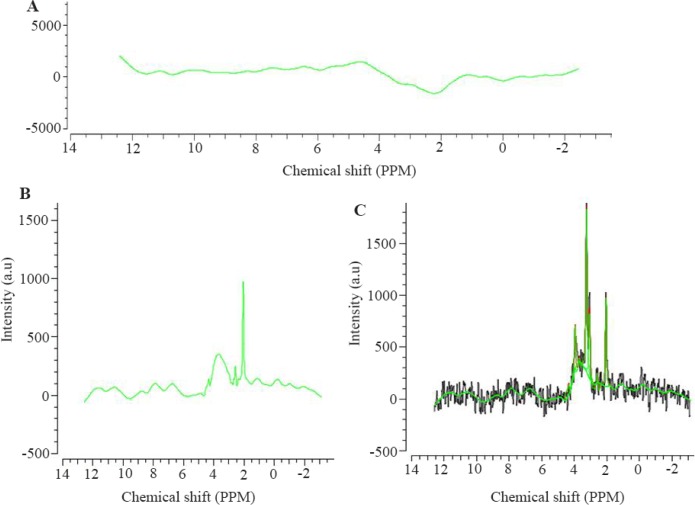
A) The baseline signal progonates from macromolecules. B) NAA signal selected as reference. C) Signal fitting is aimed at getting model signal from which concentration of metabolites can be calculated. The green curve shows the modeled signal.

The Pcho, NAA, Cr, Lac, Gln, Glu, NAAG, Taurine (Tau), and Glycine (Gly) signals were selected as reference signals, since in TE=135 *ms*, the mentioned metabolites are measurable 14. A sample of reference signals is displayed in figure 4A. Tarquin software fits (matches) the signal obtained from pre-processing stage with each reference signal individually 19. By fitting the signals, the model’s signal is obtained-the area below model curve for each metabolite is equal to its levels 19. Fitting in Tarquin software was performed using LC model algorithm 19. Figures 4B and 4C show software output after fitting. The area below the model diagram was calculated using Trapezoidal numerical integration method in Mathematics toolbox of MATLAB software.

### Statistical analysis

Normal distribution of quantitative data was evaluated for two types of voxels by Kolmogorov-Smirnov test. Normal distribution of data was analyzed by Independent Samples T-test and non-normal distribution was assessed by Mann-Whitney U test. Pearson test was used to determine probable correlation of data in patients’ voxels. Finally, sensitivity and specificity of significant analytes were determined by drawing the ROC curve. In all calculations, level of significance was determined to be p<0.05. To run statistical analysis for calculated levels from previous stage, SPSS software version 16 was used.

## Results

**Figure 5.**
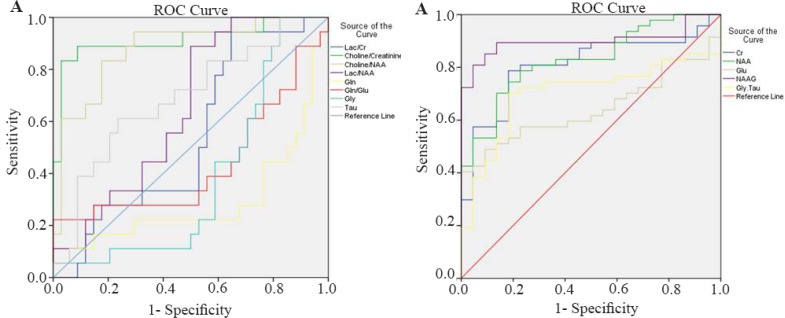
A) ROC curve for brain metabolites that were increased in tumor voxels. B) ROC curve for brain metabolites that were decreased in tumor voxels.

In this study, 5 male and 3 female (Table 1) patients with GBM were studied by MRS test, and a number of 170 tumoral and 205 normal voxels were analyzed. Given total number of normal voxels and total number of all voxels, levels of Cr, Glu, NAA, NAAG, and Gly/Tau ratio in healthy voxels were significantly higher than tumoral voxels (p=0.005, p=0.03, p<0.001, p< 0.001, and p=0.041, respectively; Table 2). In contrast, levels of Gly, Gln, Tau, Lac/Cr, Pcho/Cr, Pcho/NAA, Lac/NAA, and Gln/Glu ratios in tumoral voxels were significantly more than healthy voxels (p=0.001, p= 0.037, p<0.001, p=0.010, p<0.001, p<0.001, and p= 0.024, respectively; Table 2). However, levels of Lac and Pcho had no significant difference in the two types of voxels (Table 2). Results of correlation analysis between different biochemical parameters in tumoral voxels are shown in table 3. Finally, numerical values of sensitivity and specificity of different tests in detecting tumoral *vs*. normal voxels showed that Pcho/Cr had the highest diagnostic value with Area Under Curve (AUC) of 0.915 (sensitivity 83.3% and 97.1% specificity). The second suitable test was Pcho/NAA with AUC of to 0.879 (sensitivity 94.4% and 70.6% specificity). Third suitable test was NAAG with AUC of 0.845 (sensitivity 90.9% and 82.4% specificity) (Table 4, Figure 5).

**Table 1.** Information of the patients studied and the number of normal and tumoral voxels for each patient

**Case number**	**Sex**	**Age**	**Number of scan**	**Normal voxel**	**Tumoral voxel**
1	M	31	21	32	49
2	M	33	19	34	30
3	F	73	18	21	4
4	F	68	20	39	42
5	M	13	48	12	13
6	F	38	18	4	5
7	M	74	54	5	4
8	M	37	24	58	23

**Table 2.** The main MRS analysed parameters of tumor and normal voxels

**Parameter**	**Statistical Information**	**Tumor Voxel (n = 170)**	**Normal Voxel (n = 205)**	**p-value**
**Creatine (Cr)**	Mean±SD	45243.8±40989.9	56784.1±25570.4	0.005
**Phosphocholine (Pcho)**	Median (IQR)	62577.9 (37197.7–78336.9)	52713(35942.1–70079.7)	NS
	Range	383–382873	3145–188266	
**Glutamic Acid (Glu)**	Median (IQR)	13597 (9052.4–24418.5)	18002.6 (9811.4–33567.7)	0.030
	Range	1089–152550	2750–98338	
**Glutamine (Gln)**	Median (IQR)	10588.4 (3343.03–19059.5)	6886.6 (4034.05–12181.6)	0.037
	Range	2–142141	379–71261	
**Glycine (Gly)**	Median (IQR)	11248 (5406.1–19704.3)	8065.3 (4565.7–12307.8)	0.001
	Range	310–747186	272–78921	
**Gln/Glu Ratio**	Median (IQR)	0.43 (0.24–1.21)	0.30 (0.17–0.79)	0.024
	Range	0.00–8.85	0.00–6.33	
**Lactate (Lac)**	Median (IQR)	8433.90 (4092.6–12953)	7113.60 (3294.8–15763)	NS
	Range	270–85124	190–77207	
**Lac/Crea Ratio**	Median (IQR)	0.15 (0.08–0.37)	0.12 (0.05–0.25)	0.01
	Range	0.01–9.09	0.01–1.72	
**Pcho/Crea Ratio**	Median (IQR)	1.58 (1.18–2.39)	0.94 (0.74–1.17)	<0.001
	Range	0.04–7.34	0.31–2.82	
**N-Acetylaspartate (NAA)**	Median (IQR)	28954 (12760–57837)	57739.8 (28379.4–77123)	<0.001
	Range	2067–98759	2010–124864	
**Pcho/NAA Ratio**	Median (IQR)	1.95 (1.21–4.93)	0.93 (0.67–1.32)	<0.001
	Range	0.01–30.92	0.16–29.29	
**Lac/NAA Ratio**	Median (IQR)	0.21 (0.11–0.52)	0.13 (0.05–0.25)	<0.001
	Range	0.01–6.41	0.01–1.88	
**N-Acetylaspartylglutamic acid (NAAG)**	Median (IQR)	22160.7 (13330.5–44833)	50257.7 (28116–69125.1)	<0.001
	Range	1900–193971	865–130222	
**Taurine (Tau)**	Median (IQR)	7144.4 (3408.4–12455.7)	4911.05 (2635.6–6744.3)	<0.001
	Range	447–272522	51–32593	
**Gly/Tau Ratio**	Median (IQR)	1.21 (0.81–2.18)	1.85 (1.16–3.85)	0.041
	Range	0.31–102.66	0.08–70.86	

NS: Nonsignificant.

**Table 3.** Significant correlations between different parameters of Tumor voxels

**Parameter**	**Statistic index**	**Pcho**	**Glu**	**Gln**	**Gly**	**Gln/Glu**	**Lac**	**Lac/Cr**	**Pcho/Crea**	**NAA**	**Pcho/NAA**	**Lac/NAA**	**NAAG**	**Tau**
**Cr**	r ^a^	0.755	0.732	0.589	0.729	NS	NS	NS	−0.451	0.714	−0.26	−0.368	0.563	0.261
	P^b^	<0.001	<0.001	<0.001	<0.001				<0.001	<0.001	0.019	0.001	<0.001	0.031
**Pcho**	r	1	−0.264	NS	0.799	0.384	−0.314	−0.444	NS	0.341	NS	−0.271	NS	NS
	P		0.031		<0.001	0.002	0.007	<0.001		0.002		0.030		
**Glu**	r	-	1	0.668	0.405	NS	0.403	0.402	−0.344	0.234	NS	NS	0.712	NS
	P			<0.001	<0.001		<0.001	0.001	0.006	0.045			<0.001	
**Gln**	r	-	-	1	0.393	0.337	0.628	0.294	NS	NS	NS	0.258	0.230	NS
	P				<0.001	<0.001	<0.001	0.008				0.025	0.015	
**Gly**	r	-	-	-	1	0.312	NS	NS	NS	0.440	NS	NS	0.222	0.728
	P					0.009				<0.001			0.036	<0.001
**Gln/Glu**	r	-	-	-	-	1	0.368	NS	NS	NS	NS	NS	NS	NS
	P						0.001							
**Lac**	r	-	-	-	-	-	1	0.702	NS	NS	NS	0.307	0.269	0.429
	P							<0.001				0.004	0.005	<0.001
**Lac/Cr**	r	-	-	-	-	-	-	1	NS	NS	NS	0.265	0.235	0.515
	P											0.025	0.034	<0.001
**Pcho/Crea**	r	-	-	-	-	-	-	-	1	−0.524	0.451	0.389	−0.309	NS
	P									<0.001	<0.001	0.002	0.005	
**NAA**	r	-	-	-	-	-	-	-	-	1	−0.539	−0.426	0.375	0.304
	P										<0.001	<0.001	<0.001	0.021
**Pcho/NAA**	r	-	-	-	-	-	-	-	-	-	1	0.607	NS	NS
	P											<0.001		
**Lac/NAA**	r	-	-	-	-	-	-	-	-	-	-	1	NS	NS
	P													
**NAAG**	r	-	-	-	-	-	-	-	-	-	-	-	1	0.303
	P													0.009

NS: Non significant;

a) r coefficient;

b) p-value.

**Table 4.** Diagnostic value analysis for significant different data between tumor and normal voxel

**Parameter**	**AUC**	**Cut-off**	**Sensitivity (%)**	**Specificity (%)**
**Cr**	0.727	50456	72.7	70.6
**Glu**	0.476	11975	54.5	47.1
**Gln**	0.291	4585	44.4	23.5
**Gly**	0.381	4540	83.3	23.5
**Gln/Glu**	0.435	0.279	66.7	23.5
**Lac/Cr**	0.544	0.083	66.7	44.1
**Pcho/Cr**	0.915	1.34	83.3	97.1
**NAA**	0.759	43397	72.7	76.5
**Pcho/NAA**	0.879	0.926	94.4	70.6
**Lac/NAA**	0.650	0.847	88.9	50
**NAAG**	0.845	32059	90.9	82.4
**Tau**	0.690	0.409	83.3	44.1
**Gly/Tau**	0.674	1.14	81.8	70.6

## Discussion

In this study, for the first time, diagnostic value of detectable metabolites in long TE was investigated by simultaneous analysis of several analytes in a combination of healthy and tumoral voxels. Our results showed that, levels of Cr, Glu, NAA, NAAG, and Gly/Tau ratio in healthy voxels were significantly higher than tumoral voxels. In contrast, levels of Gly, Gln, Taurine, Lac/Cr, Pcho/Cr, Pcho/NAA, Lac/NAA, and Gln/Glu ratios in tumoral voxels were significantly higher than healthy voxels.

Different biochemical analytes have a diagnostic application in various diseases. A biomarker is a biochemical molecule measured by definite protocol to determine a pathologic process or responses to a therapeutic agent 20.

Kinoshita Y *et al* 21 reported that, concentrations of choline-containing compounds, inositol, alanine, Gly, and phosphorylethanolamine increased in glioblastoma with respect to degree of malignancy. In particular, they detected a large amount of Tau in medulloblastoma. However, they found that total Cr concentrations decreased in nonneuroectodermal tumors. Similarly, we detected lower amounts of Cr and higher concentrations of Tau in tumor voxels rather than normal ones.

Consistent with our results, Righi *et al* showed higher levels of Gly in tumor biopsies than the controls. Additionally, Gly levels significantly increased in brain metastases and GBM biopsies 22.

Different studies reported that higher activity of serine hydroxymethyltransferase or deficiency of hypoxanthine–guanine phosphoribosyltransferase in the gliomas tumor cells leads to high Gly levels. Even increased glycine peak is a differentiating agent for differentiation of glioblastoma from metastatic lesions 15.

Moreover, increased Tau is seen in medulloblastoma, pituitary microadenoma and metastatic renal cell carcinoma. In the retinoblastomas, retina has the highest levels of Tau due to its high affinity transport system 15. While, we found significant decreased ratio of Gly/Tau in tumor voxels rather than normal voxels. More studies are needed to determine role of both metabolites in glioblastoma.

However, Cr levels reduce about 15–40% in glial tumors or meningotheliomatous meningiomas. Also, in brain metastases, total Cr content is significantly lower compared to neuroectodermal tumors 15. Cr is an important cellular energy metabolism metabolite. Despite of its stability in some pathological reactions influencing central nervous system, it typically decreases in glioblastoma and astrocytomas 15,23. Possibly, higher metabolism of tumor cells leads to a decrease in the Cr levels.

Preul *et al* showed that, signal intensities of lactate, NAA, Cr, and alanine can be applied in determining various grades of glioma 24. Our results showed that, NAA levels decreased in tumoral voxels compared to normal ones. NAA as the second most abundant amino acid in the brain markedly decreases or is absent in malignant or benign tumors involving axonal loss. Apparently, absence of NAA biosynthetic enzyme (aspartate N acetyltransferase) in brain tumors is main cause of NAA signal loss 15.

NAA is one of the most abundant amino acid derivatives in the brain and is considered as a source of metabolic acetate in brain cells. Levels of both NAA and NAAG decreased in glioma tumors. It is assumed that NAA hydrolysis provides high levels of acetate molecules used for lipogenesis in glioma tumor cells 25.

Lower levels of NAAG were found in tumoral voxels rather than normal voxels. This molecule is formed in neurons from NAA by adding glutamate residue and is degraded only in astrocytes. Both NAA and NAAG have important role in cell-specific glial signaling. They are important in function and development of brain, regulation of interactions of brain cells, maintenance of nervous system, and even as a neurotransmitter in CNS acting as a partial agonist of N-methyl-d- aspartate receptors. NAAG has an essential role in glial cell metabolism. In addition, NAAG aciduria has been detected in Canavan disease as neurodegenerative disease 26,27. Recently, a study suggested that astrocytes, oligodendrocyte progenitor cells, and neural stem cells could all serve as the cell of origin for glioblastoma occurrence 27. Probably, decreased level of NAAG has a causative metabolic and signaling effect on glioblastoma development.

Simultaneously, lower levels of glutamate and higher levels of glutamine were found in tumoral voxels compared to normal voxels. In this line, NAAG can also serve as a cellular storage for glutamic acid. On the other hand, Glu is converted into Gln returning to neurons by astrocytes 26. This reaction may lead to decreased Glu levels and increased Gln levels.

Furthermore, a part of Glu enters to Krebs cycle *via* converting to α-ketoglutarate for ATP production 28. Increased level of Gln in glioblastoma is possibly due to higher energy demand of tumor cell 29 and higher activity of glutamine synthase. Apparently, increased uptake of glutamine and its changing to glutamate is an important process in highly proliferated tumor cells 30.

Our results showed that Pcho level was the same between tumoral and normal voxels. Nevertheless, two different studies have reported that, Choline Kinase in several brain tumors is overexpressed and increased choline peak reflects increased cell membrane synthesis, turnover, and integrity 15. Nelson *et al* indicated that an increase in the choline and a decrease in the NAA is so important to determine spatial extent of metabolic abnormality and consequent tumor activity 31. Similarly, another study revealed a significant decrease in the NAA signal intensity in diaschitic cerebellar hemisphere and an insignificant decrease in the choline and Cr signal intensities.

Possibly, increased levels of choline are associated with cell proliferation, while NAA is a marker for density and viability of neurons 32. More studies are required to clarify exact role of choline changes in tumor metabolism.

In our study, lactate levels of both tumoral and normal voxels were similar. However, increased rates of lactate production were reported. Also, lactate level was found to be associated with a range of tumors and tumor grade or tumor metabolic activity. In this line, lactate peak was found to be usually absent in low -grade brain tumors. It increases in high-grade primary brain tumor 15. Likely, this difference may be due to differences in ethnicity or our small sample size.

Different reduced or increased ratios of glioblastoma biomarkers have pivotal role in diagnosis. In another study 33, in recurrent glioma tumor, Pcho/NAA and Pcho/Cr ratios were significantly higher than in radiation injury. However, NAA/Cr ratio was lower in recurrent glioma tumor than in radiation injury. Besides, in radiation injury, Pcho/Cr and Pcho/NAA ratios were significantly higher compared to normal-appearing white matter, and NAA/Cr ratio was lower in radiation injury than in normal-appearing white matter.

Tumoral voxels in our study were found to have higher NAA/Pcho compared to normal voxels, which is consistent with some studies 23,32,34,35. Pcho/NAA has been described as suitable biomarker of tumor prognosis and monitoring of treatment response. Similarly, Bulik *et al* illustrated that in recurrence glioblastoma at N-acetylaspartate ≤1.5 *mM*, Pchoe/N-acetylaspartate ≥1.4, both sensitivity and specificity were equal to 100 and 91.7%, respectively 34.

Receiver operating characteristic analyses performed in the present study for assessment of NAA/Pcho ratio revealed that at 0.922 as cut-off, AUC was equal to 0.879 and sensitivity and specificity were equal to 94.4 and 70.6%, respectively. Ratai *et al* reported lower AUC in tumor (AUC=0.83) and higher AUC in peritumoral regions (AUC=0.95) 23. However, Tomas *et al* 32 reported on Pcho/NAA ratio with highest significant sensitivity and specificity for glioblastoma recurrence (AUC=0.993; sensitivity of 100.0% and specificity of 94.7%). Furthermore, they reported on Pcho/Cr ratio with a weak sensitivity and specificity (74.6 and 63.2%, respectively; AUC=0.691) 32. In contrast, our results demonstrated that, assessment of Pcho/Cr ratio was the best test for differentiation between normal and tumoral voxels. It had AUC equal to 0.915 at 1.34 as cut-off with 83.3 and 97.1% as sensitivity and specificity, respectively. Nevertheless, Ando *et al* 36 reported that, despite of significant higher ratio of Pcho/Cr in cases with residual/recurrent tumors than in non-neo-plastic lesions; at point of 1.5 as cut-off, its sensitivity and specificity was equal to 64 and 83%, respectively. Presumably, this difference in sensitivity and specificity results of AUC is related to different technical details and differences in studied sample size.

Finally, in a systematic review, seven studies were reviewed including a total of 261 patients with high -grade glioma 37. Analysis showed that in peritumoral tissue pooled sensitivity/specificity of Pcho/NAA and Pcho/Cr ratio were equal to 0.85/0.93 and 0.86/0.86, respectively. Likewise, Pcho/NAA ratio had higher value of AUC and higher specificity rather than Pcho/Cr ratio in peritumoral region. They suggested that Pcho/NAA ratio of peritumoral region should be applied to increase accuracy of MRS to differentiate high-grade gliomas from metastases.

Ratai *et al* 23 reported that, Lac/Cr ratio in tumor was an important negative predictor of 6-month progression free survival. They also demonstrated that poorer outcome was associated with higher Lac/Cr. (AUC=0.79) 23. Our results indicated that, Lac/Cr ratio AUC was equal to 0.544, showing this index is weaker biomarker for glioblastoma rather than Pcho/NAA.

There were some limitations in our study. Following up the patients was not possible because, they often did not refer again to the center. In addition, sample size of the study was small. However, the subjects were selected during 18 months. Certainly, similar studies in larger scale and with larger sample size could be useful for better determination of ^1^H-MRS power to detect GBM.

## Conclusion

In summary, results of the current study showed that, compared to patients with glioblastoma with ^1^HMRS, Pcho/Cr and Pcho/NAA ratios, and NAAG are most important and useful parameters to differentiate between tumoral and normal voxels. Further studies are needed to determine ^1^H-MRS ability to differentiate brain tumor region from healthy one.

## References

[1] Tome-Garcia J Tejero R Nudelman G Yong RL Sebra R Wang H Prospective isolation and comparison of human germinal matrix and glioblastoma EGFR+ populations with stem cell properties. Stem Cell Reports 2017;8(5):1421–1429. 28434940 10.1016/j.stemcr.2017.03.019 PMC5425658

[2] Al-Saffar NM Marshall LV Jackson LE Balarajah G Eykyn TR Agliano A Lactate and choline metabolites detected in vitro by nuclear magnetic resonance spectroscopy are potential metabolic biomarkers for PI3K inhibition in pediatric glioblastoma. PLoS One 2014;9(8):e103835. 10.1371/journal.pone.0103835 PMC4118961 25084455

[3] Burlina AP Aureli T Bracco F Conti F Battistin L. MR spectroscopy: a powerful tool for investigating brain function and neurological diseases. Neurochem Res 2000;25(9–10):1365–1372. 11059807 10.1023/a:1007660632520

[4] Smirniotopoulos JG Murphy FM Rushing EJ Rees JH Schroeder JW. Patterns of contrast enhancement in the brain and meninges. Radiographics 2007;27(2):525–551. 17374867 10.1148/rg.272065155

[5] Parra NA Maudsley AA Gupta RK Ishkanian F Huang K Walker GR Volumetric spectroscopic imaging of glioblastoma multiforme radiation treatment volumes. Int J Radiat Oncol Biol Phys 2014;90(2):376–384. 25066215 10.1016/j.ijrobp.2014.03.049 PMC4346247

[6] Xia Y Yang C Hu N Yang Z He X Li T Exploring the key genes and signaling transduction pathways related to the survival time of glioblastoma multiforme patients by a novel survival analysis model. BMC Genomics 2017;18(Suppl 1):950. 28198665 10.1186/s12864-016-3256-3 PMC5310279

[7] Crain ID Elias PS Chapple K Scheck AC Karis JP Preul MC. Improving the utility of 1H-MRS for the differentiation of glioma recurrence from radiation necrosis. J Neurooncol 2017;133(1):97–105. 28555423 10.1007/s11060-017-2407-y

[8] Ulmer S Backens M Ahlhelm FJ. Basic principles and clinical applications of magnetic resonance spectroscopy in neuroradiology. J Comput Assist Tomogr 2016;40(1): 1–13. 26484954 10.1097/RCT.0000000000000322

[9] Haaga JR Boll D. Smith Paul CT and MRI of the Whole Body, 2-Volume Set. 5th ed. Dogra V Forsting M Gilkeson R Ha KH Sundaram M (eds). Philadelphia: Mosby, Elsevier; 2009 2904 p.

[10] Azarakhsh F Changizi V. Metabolites role in detecting brain tumors using nuclear magnetic resonance spectroscopy and comparing their densities in tumoral patients with those in healthy individuals. J Payavard Salamat 2016;10(3):248–257.

[11] Einstein DB Wessels B Bangert B Fu P Nelson AD Cohen M Phase II trial of radiosurgery to magnetic resonance spectroscopy–defined high-risk tumor volumes in patients with glioblastoma multiforme. Int J Radiation Oncol Biol Phys 2012;84(3):668–674. 10.1016/j.ijrobp.2012.01.020 PMC4334318 22445005

[12] Deviers A Ken S Filleron T Rowland B Laruelo A Catalaa I Evaluation of the lactate-to-N-acetylaspartate ratio defined with magnetic resonance spectroscopic imaging before radiation therapy as a new predictive marker of the site of relapse in patients with glioblastoma multiforme. Int J Radiat Oncol Biol Phys 2014; 90(2):385–393. 25104068 10.1016/j.ijrobp.2014.06.009

[13] Parto Dezfouli MA Parto Dezfouli M Ahmadian A Frangi AF Esmaeili Rad M Saligheh Rad H. Quantification of 1H–MRS signals based on sparse metabolite profiles in the time-frequency domain. NMR Biomed 2017; 30(2). 10.1002/nbm.3675 28052436

[14] Bottomley PA Griffiths JR. Handbook of magnetic resonance spectroscopy in vivo: MRS theory, practice and applications. USA: John Wiley & Sons; 2016 1232 p.

[15] Verma A Kumar I Verma N Aggarwal P Ojha R. Magnetic resonance spectroscopy-revisiting the biochemical and molecular milieu of brain tumors. BBA Clin 2016; 5:170–178. 27158592 10.1016/j.bbacli.2016.04.002 PMC4845155

[16] Kreis R. Issues of spectral quality in clinical 1H-magnetic resonance spectroscopy and a gallery of artifacts. NMR Biomed 2004;17(6):361–381. 15468083 10.1002/nbm.891

[17] Cianfoni A Law M Re TJ Dubowitz DJ Rumboldt Z Imbesi SG. Clinical pitfalls related to short and long echo times in cerebral MR spectroscopy. J Neuroradiol 2011; 38(2):69–75. 21215455 10.1016/j.neurad.2010.10.001

[18] Bertholdo D Watcharakorn A Castillo M. Brain proton magnetic resonance spectroscopy: introduction and overview. Neuroimaging Clin N Am 2013;23(3):359–380. 23928194 10.1016/j.nic.2012.10.002

[19] Wilson M Reynolds G Kauppinen RA Arvanitis TN Peet AC. A constrained least-squares approach to the automated quantitation of in vivo 1H magnetic resonance spectroscopy data. Magn Reson Med 2011;65(1):1–12. 20878762 10.1002/mrm.22579

[20] Biomarkers definitions working group Biomarkers and surrogate endpoints: preferred definitions and conceptual framework. Clin Pharmacol Ther 2001;69(3):89–95. 11240971 10.1067/mcp.2001.113989

[21] Kinoshita Y Yokota A. Absolute concentrations of metabolites in human brain tumors using in vitro proton magnetic resonance spectroscopy. NMR Biomed 1997; 10(1):2–12. 9251109 10.1002/(sici)1099-1492(199701)10:1<2::aid-nbm442>3.0.co;2-n

[22] Righi V Andronesi OC Mintzopoulos D Black PM Tzika AA. High-resolution magic angle spinning magnetic resonance spectroscopy detects glycine as a biomarker in brain tumors. Int J Oncol 2010;36(2):301–306. 20043062 10.3892/ijo_00000500 PMC3715372

[23] Ratai EM Zhang Z Fink J Muzi M Hanna L Greco E ACRIN 6684: multicenter, phase II assessment of tumor hypoxia in newly diagnosed glioblastoma using magnetic resonance spectroscopy. PLoS One 2018;13(6): e0198548. 10.1371/journal.pone.0198548 PMC6002091 29902200

[24] Preul M Caramanos Z Collins DL Villemure JG Leblanc R Olivier A Accurate non-invasive diagnosis of human brain tumors by using proton magnetic resonance spectroscopy. Nat Med 1996;2:323–325. 8612232 10.1038/nm0396-323

[25] Long PM Moffett JR Namboodiri AM Viapiano MS Lawler SE Jaworski DM. N-acetylaspartate (NAA) and N-acetylaspartylglutamate (NAAG) promote growth and inhibit differentiation of glioma stem-like cells. J Biol Chem 2013;288(36):26188–26200. 23884408 10.1074/jbc.M113.487553 PMC3764823

[26] Baslow MH. Functions of N-acetyl-L-aspartate and N-acetyl-L-aspartylglutamate in the vertebrate brain: role in glial cell-specific signaling. J Neurochem 2000;75(2): 453–459. 10899919 10.1046/j.1471-4159.2000.0750453.x

[27] Zong H Parada LF Baker SJ. Cell of origin for malignant gliomas and its implication in therapeutic development. Cold Spring Harb Perspect Biol 2015;7(5). pii: a020610. 10.1101/cshperspect.a020610 PMC4448618 25635044

[28] Maus A Peters GJ. Glutamate and α-ketoglutarate: key players in glioma metabolism. Amino Acids 2017;49(1): 21–32. 27752843 10.1007/s00726-016-2342-9 PMC5241329

[29] Elsakka AMA Bary MA Abdelzaher E Elnaggar M Kalamian M Mukherjee P Management of glioblastoma multiforme in a patient treated with ketogenic metabolic therapy and modified standard of care: a 24-month follow-up. Front Nutr 2018;5:20. 29651419 10.3389/fnut.2018.00020 PMC5884883

[30] Liubinas SV O’Brien TJ Moffat BM Drummond KJ Morokoff AP Kaye AH. Tumour associated epilepsy and glutamate excitotoxicity in patients with gliomas. J Clin Neurosci 2014;21(6):899–908. 24746886 10.1016/j.jocn.2014.02.012

[31] Nelson SJ. Multivoxel magnetic resonance spectroscopy of brain tumors. Mol Cancer Ther 2003;2(5):497–507. 12748312

[32] Kazd T Bulik M Pospisil P Lakomy R Smrcka M Slampa P Advanced MRI increases the diagnostic accuracy of recurrent glioblastoma: Single institution thresholds and validation of MR spectroscopy and diffusion weighted MR imaging. Neuroimage Clin 2016;11: 316–321. 27298760 10.1016/j.nicl.2016.02.016 PMC4893011

[33] Zeng QS Li CF Zhang K Liu H Kang XS Zhen JH. Multivoxel 3D proton MR spectroscopy in the distinction of recurrent glioma from radiation injury. J Neurooncol 2007;84(1):63–69. 17619225 10.1007/s11060-007-9341-3

[34] Bulik M Kazda T Slampa P Jancalek R. The diagnostic ability of follow-up imaging biomarkers after treatment of glioblastoma in the temozolomide era: implications from proton MR spectroscopy and apparent diffusion coefficient mapping. Biomed Res Int 2015;2015:641023. 10.1155/2015/641023 PMC4584055 26448943

[35] Roldan-Valadez E Rios C Motola-Kuba D Matus-Santos J Villa AR Moreno-Jimenez S. Choline-to-N-acetyl aspartate and lipids-lactate-to-creatine ratios together with age assemble a significant Cox’s proportional-hazards regression model for prediction of survival in high-grade gliomas. Br J Radiol 2016;89(1067):20150502. 10.1259/bjr.20150502 PMC5124820 27626830

[36] Ando K Ishikura R Nagami Y Morikawa T Takada Y Ikeda J [Usefulness of Cho/Cr ratio in proton MR spectroscopy for differentiating residual/recurrent glioma from non-neoplastic lesions]. Nihon Igaku Hoshasen Gakkai Zasshi 2004;64(3):121–126. Japanese. 15148787

[37] Wang Q Zhang J Xu W Chen X Zhang J Xu B. Role of magnetic resonance spectroscopy to differentiate high-grade gliomas from metastases. Tumour Biol 2017;39(6): 1010428317710030. 10.1177/1010428317710030 28631566

